# Predicting orchiectomy in testicular torsion using hybrid machine learning and explainable AI: a web-based clinical decision support system

**DOI:** 10.1007/s00345-026-06448-4

**Published:** 2026-06-08

**Authors:** Ali Çift, Hüseyin Kutlu, Ferhat Çoban, Hasan Sulhan, Sait Sever, Mustafa Kemal Koç, Mustafa Kılınç, Bedreddin Kalyenci

**Affiliations:** 1 Faculty of Medicine, Department of Urology, Adıyaman University, Adıyaman, Turkey; 2 Faculty of Medicine, Department of Biostatistics and Medical Informatics, Adıyaman University, Adıyaman, Turkey; 3 Faculty of Medicine, Adıyaman University Central Campus, Dean’s Office Altınşehir/Center/Adıyaman, Adıyaman, Turkey

**Keywords:** Testicular torsion, Orchiectomy, Explainable artificial intelligence, SHAP, Nomogram, Web application

## Abstract

**Objective:**

To develop a high-accuracy prediction model using hybrid machine learning (ML) and explainable artificial intelligence (XAI) techniques for distinguishing between orchiectomy and detorsion in testicular torsion (TT), and to create an interactive web application and nomogram for clinical use.

**Materials and methods:**

Data from 117 patients who underwent surgical treatment for TT at our clinic between January 2005 and June 2025 were retrospectively analyzed (detorsion: 83, orchiectomy: 34). From twenty initial features, seven optimal features were selected using a hybrid Particle Swarm Optimization-Grey Wolf Optimization (PSO-GWO) algorithm. Class imbalance was addressed using SVMSMOTE. Four ensemble learning algorithms were compared, and CatBoost was selected as the final model. SHAP, LIME, Partial Dependence Plot (PDP), and Individual Conditional Expectation (ICE) analyses were performed for model interpretability. A seven-feature nomogram was developed using logistic regression. A two-module web application was created using Python Dash.

**Results:**

PSO-GWO identified seven optimal features (Age, Symptom Duration, PDW, PCT, MPV, MLR, SII). CatBoost demonstrated the highest performance (AUC: 0.923, Accuracy: 89.5%). XAI analyses identified symptom duration as the strongest predictor (SHAP: 52.72%), followed by MLR (14.24%) and PDW (12.09%). PDW emerged as a novel biomarker with minimal prior investigation in the literature yet demonstrated strong discriminatory power (inverse relationship, cut-off < 17.9 fL). PDP analysis revealed a distinct inflection point at approximately 7 h, corroborating the ROC cut-off values. ICE analysis revealed substantial individual heterogeneity (score > 0.36). The nomogram demonstrated good discrimination (AUC: 0.818) and excellent calibration (Hosmer-Lemeshow *p* = 0.556). A simplified three-feature risk scoring system (0–3 points) was developed. The web application (medicalinformaticsttrc.adiyaman.edu.tr) provided real-time risk calculation (< 50 ms).

**Conclusion:**

The CatBoost model developed with hybrid PSO-GWO algorithm and XAI techniques achieved high accuracy in predicting orchiectomy in TT. PDW was identified as a novel, practical, and cost-effective biomarker. The developed web application and risk scoring system may support preoperative counseling, surgical preparedness, and clinical decision-making in the emergency department. The “detorsion” outcome indicates intraoperative non-removal of the testis and does not confirm long-term testicular viability. This exploratory model requires external validation and multicenter prospective studies before clinical adoption.

**Supplementary Information:**

The online version contains supplementary material available at 10.1007/s00345-026-06448-4.

## Introduction

Testicular torsion (TT) is a urological emergency characterized by reduced testicular blood flow resulting from rotation of the spermatic cord around its own axis, with an increasing incidence over the past two decades and requiring urgent intervention, occurring with an incidence of 1/4000 in males under 25 years of age. The most serious complication is testicular necrosis, which may result in orchiectomy and affect future fertility [1]. Irreversible changes and necrosis in TT are time-dependent, with a critical window of approximately four to six hours. Therefore, rapid diagnosis is essential. Urological history, clinical findings, and the Testicular Workup for Ischemia and Suspected Torsion (TWIST) score can be used to identify TT. Doppler ultrasonography (US) can contribute to the diagnosis of TT provided it does not cause delays in surgical exploration, and has become the standard imaging modality for patients with suspected TT. However, these diagnostic parameters do not always provide clear information regarding testicular viability. Treatment of TT requires emergency surgical intervention, and the surgical approach (detorsion or orchiectomy) depends on testicular viability [2, 3]. Preoperative prediction of testicular viability enables clinicians to be informed and prepared prior to surgery, and allows more reliable information to be provided to patients and their family members. It may also enable families to optimize their expectations regarding postoperative outcomes and may prevent medicolegal issues.

The existing literature includes studies, predominantly conducted using conventional statistical methods, reporting that testicular viability can be predicted using symptom duration, degree of torsion, hematological parameters, and Doppler US [4–8]. The reliance on statistical methods for variable selection limits the capacity to effectively process multiple variables and nonlinear structures, thereby adversely affecting predictive performance.

In recent years, machine learning (ML)-based approaches have emerged as promising alternatives for surgical outcome prediction, demonstrating superior predictive power compared to classical statistical methods [9]. Explainable artificial intelligence (XAI) techniques can transparently reveal which variables ML models rely upon for predictions. Thus, not only is predictive accuracy enhanced, but the acceptability of clinical decision support systems is also strengthened.

Visual and user-friendly tools are critically important for integrating developed ML models into clinical practice. Nomograms are evidence-based tools that convert complex prediction models into simple, comprehensible, and bedside-applicable graphic formats, enabling clinicians to perform manual calculations. In modern medical practice, there is an increasing need for digital platforms to transform ML models into clinical decision support systems. Web-based applications can provide clinicians with real-time risk stratification by performing complex algorithmic calculations instantaneously.

In this study, we aimed to use ML algorithms to predict testicular viability and estimate orchiectomy requirement in the preoperative period in patients with TT, and to develop a clinically applicable, reliable, and innovative decision support tool by making these models interpretable using XAI techniques. Our study is the first to utilize XAI techniques for determining testicular viability in patients with TT.

## Materials and methods

### Ethical approval

Ethical approval for this study was obtained from the Ethics Committee of Adıyaman University Faculty of Medicine (Approval number: 2025/9–15). Datafrom 117 patients who were surgically treated and followed up with a diagnosis of TT at our clinic between January 2005 and June 2025 were retrospectively analyzed. The diagnosis of TT was established based on history, physical examination, routine blood tests, and Doppler US. Patients with missing data, neonatal torsion, initially treated conservatively but ultimately could not be saved, negative scrotal exploration, or torsion of the testicular appendix were excluded, as were those with autoimmune diseases that could alter complete blood count (CBC) parameters, those regularly using non-steroidal anti-inflammatory drugs or systemic steroids, those with a history of radiotherapy or chemotherapy, and those with acute viral or bacterial infections.

All operations were performed under general or spinal anesthesia in the supine position following prophylactic antibiotic therapy. Immediately after surgical detorsion, a warm moist gauze was placed over the testis, and the testis was kept warm for 15–20 min. When testicular reperfusion was observed, fixation was performed and the testis was placed in the scrotum. Orchiectomy was performed in cases of black, necrotic, or non-perfused testis. The contralateral testis was routinely subjected to fixation. Patients were divided into two groups according to the type of surgical intervention: the detorsion group and the orchiectomy group.

Clinical and laboratory data were collected retrospectively from electronic medical records. The dataset contained 16 basic features: age as a demographic variable, symptom duration (hours) as a clinical parameter, and 14 hematological parameters. Complete blood count indices included white blood cell count (WBC), red cell distribution width (RDW), platelet count, platelet distribution width (PDW), plateletcrit (PCT), and mean platelet volume (MPV). All features were continuous variables. The dataset was examined for missing values; no missing data were identified across any of the 16 features for all 117 patients (complete case rate: 100%). Patients with incomplete medical records were excluded during the initial data extraction phase as specified in the exclusion criteria.

### Statistical analysis

The normality of continuous variables was assessed using the Shapiro-Wilk test. Since most variables did not follow a normal distribution, the Mann-Whitney U test was used for group comparisons. Benjamini-Hochberg FDR correction was applied for Type I error control. Effect sizes were calculated using Cliff’s Delta (|δ|) (small: 0.147, medium: 0.330, large: 0.474). 95% confidence intervals for median differences were calculated using the bootstrap method. Statistical significance was set at p_FDR < 0.05. Analyses were performed using Python 3.12 (SciPy 1.11).

### Feature selection and model development

The initial feature pool comprised 16 preoperatively available variables: patient age, symptom duration, and 14 routine CBC parameters. Variables were selected based on three criteria: (1) universal availability in the emergency setting without additional cost or delay, (2) biological plausibility in the context of testicular ischemia–reperfusion injury (inflammation, platelet activation, oxidative stress), and (3) prior literature support for CBC-derived indices in testicular torsion outcome prediction (6).

A hybrid Particle Swarm Optimization-Grey Wolf Optimization (PSO-GWO) algorithm was used to identify the most discriminative features for predicting surgical outcome. The Matthews Correlation Coefficient (MCC) obtained from 10-fold cross-validation with a class-weighted balanced Random Forest classifier was used as the fitness function. Algorithm parameters were set as follows: 30 particles, 50 iterations, and hybridization factor α = 0.5.

The PSO-GWO approach was compared with nine established methods: LASSO, ElasticNet, BORUTA, Recursive Feature Elimination (RFE), Chi-square test, ANOVA F-test, Mutual Information, Neighborhood Rough Set (NRS), and CBR-BGOA. All methods were evaluated using identical 10-fold stratified cross-validation.

### Sampling strategies for imbalanced dataset

Eight different synthetic data generation methods were compared to address class imbalance in the dataset (detorsion: *n* = 83, orchiectomy: *n* = 34, detorsion: orchiectomy = 2.44:1). The original dataset was divided into 80% training (*n* = 93) and 20% test (*n* = 24) sets using stratified sampling. The test set was not subjected to any manipulation and was used solely for model performance evaluation.

The compared methods were: (1) No sampling (baseline), (2) Random Oversampling, (3) SMOTE (Synthetic Minority Over-sampling Technique), (4) BorderlineSMOTE, (5) SVMSMOTE, (6) ADASYN (Adaptive Synthetic Sampling), (7) SMOTE-ENN (SMOTE + Edited Nearest Neighbors), and (8) SMOTE-Tomek. A k = 5 nearest neighbors parameter was used for all SMOTE-based methods.

For each method, a model was trained using a Random Forest classifier (100 trees, maximum depth = 5), and performance was evaluated on the original test set. The Matthews Correlation Coefficient (MCC) was used as the primary performance metric.

### Machine learning model development

Four ensemble learning algorithms were evaluated for predicting testicular torsion surgical outcomes: CatBoost (200 iterations), LightGBM, Gradient Boosting, and Bagging. Model performances were evaluated using 10-fold stratified cross-validation.

All features were standardized using z-score normalization (mean = 0, standard deviation = 1) prior to model training. Performance metrics including accuracy, AUC, sensitivity, specificity, positive predictive value (PPV), negative predictive value (NPV), F1-score, and Matthews correlation coefficient (MCC) were calculated. MCC was used as the priority metric for model selection because it measures balanced classification quality in imbalanced datasets. 95% confidence intervals for all metrics were calculated using the standard error method from 10-fold cross-validation results.

### Explainable artificial intelligence analysis

Five different explainable artificial intelligence (XAI) methods were applied to the best-performing model: (a) SHAP (SHapley Additive exPlanations), (b) LIME (Local Interpretable Model-agnostic Explanations), (c) model-based feature importance, (d) permutation-based feature importance, and (e) PDP-ICE analysis.

SHAP analysis was selected as the primary interpretability method due to its mathematical rigor based on game theory foundations and consistency guarantees. SHAP values were calculated for all test samples using TreeExplainer, and global feature importance was determined by averaging the absolute SHAP values.

The LIME method provided local explanations by generating 1000 perturbation samples for each test sample. Global ranking was obtained by averaging the LIME importance scores across all test samples.

Model-based feature importance was extracted directly from the internal mechanism of the CatBoost algorithm.

Scikit-learn Partial Dependence Display was used for Partial Dependence Plot (PDP) and Individual Conditional Expectation (ICE) analysis. One-dimensional PDP was calculated for each feature, and two-dimensional PDP was calculated for strong interactions. Individual heterogeneity was assessed using ICE plots. Non-parallel ICE curves indicated feature interactions.

Ranking standard deviations were calculated to assess consistency across methods: σ < 1.0 was defined as high agreement, 1.0 ≤ σ < 2.0 as moderate agreement, and σ ≥ 2.0 as low agreement.

An 80 − 20 stratified single data split was used for XAI analyses, and SVMSMOTE was applied only to the training set.

### Optimal threshold values and risk stratification

Optimal cut-off points (Youden Index) were determined for the selected features using ROC curve analysis for use in the model. Two risk scoring systems were developed: (a) Comprehensive 7-feature score (0–6 points, orchiectomy rates: score 0–1: ~0%, score 2: ~5%, score 3: ~44%, score 4: ~75%, score 5–6: 100%), and (b) Simple 3-feature score (0–3 points, for bedside use). Meeting each criterion was scored as 1 point.

### Nomogram and web application development

The nomogram was developed as a separate logistic regression model, independent of the CatBoost classification model, with the seven selected features (penalty=‘l2’, C = 1.0, max_iter = 2000, random_state = 42). Logistic regression was deliberately chosen because nomograms require a linear, additive scoring framework to enable manual point calculation, which is incompatible with nonlinear ensemble methods such as CatBoost. Both models utilized the same seven PSO-GWO-selected features. The lower discriminative performance of the nomogram (AUC: 0.818) compared to CatBoost (AUC: 0.923) represents the expected trade-off between model complexity and bedside interpretability. A two-module web application was developed using the Python Dash framework (v2.14).

## Results

### Univariate analysis

Clinical and laboratory parameters were compared between the detorsion (*n* = 83) and orchiectomy (*n* = 34) groups using the Mann-Whitney U test. Shapiro-Wilk normality testing revealed that only two of the 16 variables (RDW and MPV) exhibited normal distribution (*p* > 0.05). Therefore, non-parametric tests were preferred for all comparisons. Benjamini-Hochberg False Discovery Rate (FDR) correction was applied to control for multiple comparison error.

Following FDR correction, four of the 16 variables showed statistically significant differences between groups (Table 1). The strongest discriminating parameter was symptom duration, which was markedly longer in the orchiectomy group (p_FDR < 0.001, Cliff’s δ = 0.719). Among inflammatory markers, monocyte count (p_FDR = 0.011, δ = 0.377) and monocyte-to-lymphocyte ratio (MLR) (p_FDR = 0.016, δ = 0.340) were significantly higher in the orchiectomy group. As an unexpected finding, platelet distribution width (PDW) was significantly lower in the orchiectomy group compared to the detorsion group (p_FDR = 0.016, δ = −0.343). Notably, NLR (p_FDR = 0.947, Cliff’s δ = 0.008) and PLR (p_FDR = 0.558, δ = 0.111) demonstrated no significant intergroup difference, suggesting limited independent discriminatory value in this cohort.

**Table 1:** Univariate Comparison of Clinical and Laboratory Parameters Between Treatment Groups

Variable	Detorsion (*n* = 83) Mdn (IQR)	Orchiectomy (*n* = 34) Mdn (IQR)	Difference^a^	95% CI^b^	U	*p*	*p*_FDR_	Cliff’s δ
Symptom Duration (h)	3.00 (2.00–6.00)	9.00 (8.00–12.00)	+ 6.00	(+ 4.50, + 9.00)	396.5	< 0.001	< 0.001	+ 0.719
Monocyte (×10³/µL)	0.54 (0.45–0.69)	0.79 (0.50–1.04)	+ 0.25	(+ 0.05, + 0.38)	879.0	0.001	0.011	+ 0.377
PDW (fL)	17.00 (13.70–18.96)	15.35 (11.22–16.75)	−1.65	(− 4.60, − 0.15)	1,894.5	0.004	0.016	−0.343
MLR	0.24 (0.19–0.34)	0.39 (0.23–0.48)	+ 0.15	(+ 0.05, + 0.20)	931.0	0.004	0.016	+ 0.340
PIV	489.89 (269.94–819.44)	745.84 (395.75–1,366.62)	+ 255.94	(− 4.92, + 768.00)	1,033.0	0.023	0.075	+ 0.268
RDW (%)	12.60 (11.82–13.50)	13.50 (12.43–13.95)	+ 0.90	(+ 0.15, + 1.24)	1,049.5	0.030	0.080	+ 0.256
WBC (×10³/µL)	9.75 (8.48–12.21)	11.58 (8.57–13.33)	+ 1.83	(− 0.59, + 3.15)	1,186.0	0.178	0.316	+ 0.159
NLR	2.85 (1.77–5.25)	3.24 (1.77–5.56)	+ 0.39	(− 1.40, + 1.77)	1,399.5	0.947	0.947	+ 0.008
PLR	117.49 (89.31–169.45)	138.79 (96.61–195.26)	+ 21.30	(− 9.95, + 49.82)	1,254.5	0.349	0.558	+ 0.111
SII	879.20 (496.60–1,409.12)	1,035.78 (501.24–1,614.68)	+ 156.58	(− 253.46, + 732.03)	1,294.0	0.484	0.605	+ 0.083
Neutrophil (×10³/µL)	6.95 (5.05–9.15)	7.50 (4.70–10.06)	+ 0.55	(− 1.65, + 2.62)	1,383.0	0.869	0.947	+ 0.020
Age (years)	17.00 (15.00–20.00)	16.00 (13.00–20.75)	−1.00	(− 3.00, + 1.50)	1,550.5	0.403	0.586	−0.099

Values are presented as Mdn (IQR). Mdn = Median; IQR = Interquartile Range (Q1–Q3); CI = 95% Bootstrap Confidence Interval (10,000 resamples, percentile method); U = Mann-Whitney U statistic; p = two-sided uncorrected p-value; p_FDR_ = Benjamini-Hochberg False Discovery Rate adjusted p-value. Variables reaching p_FDR_< 0.05 are displayed in bold

^a^ Difference = Median(Orchiectomy) − Median(Detorsion). A positive value indicates a higher value in the orchiectomy group; a negative value indicates a higher value in the detorsion group (e.g., PDW, Age)

^b^ 95% Bootstrap CI for the median difference (Orchiectomy − Detorsion)

Cliff’s δ: |δ|< 0.147 = negligible; 0.147–0.330 = small; 0.330–0.474 = medium; > 0.474 = large (Romano et al., 2006). Positive δ indicates higher values in the orchiectomy group; negative δ indicates lower values in the orchiectomy group

### Feature selection and model performance

The PSO-GWO hybrid algorithm demonstrated the highest performance among all feature selection methods tested (Supplementary Table S2), achieving a mean MCC value of 0.705 ± 0.092 with seven features. The algorithm converged rapidly, reaching optimal performance within the first 10 iterations.

### Imbalanced dataset management and performance improvement

Eight different sampling strategies were compared (Supplementary Table S3). The SVMSMOTE method demonstrated the highest performance among all alternatives (MCC = 0.713). The SVMSMOTE method showed a 49.7% improvement in MCC compared to the baseline model without sampling.

### Model performance comparison

**Fig. 1:**
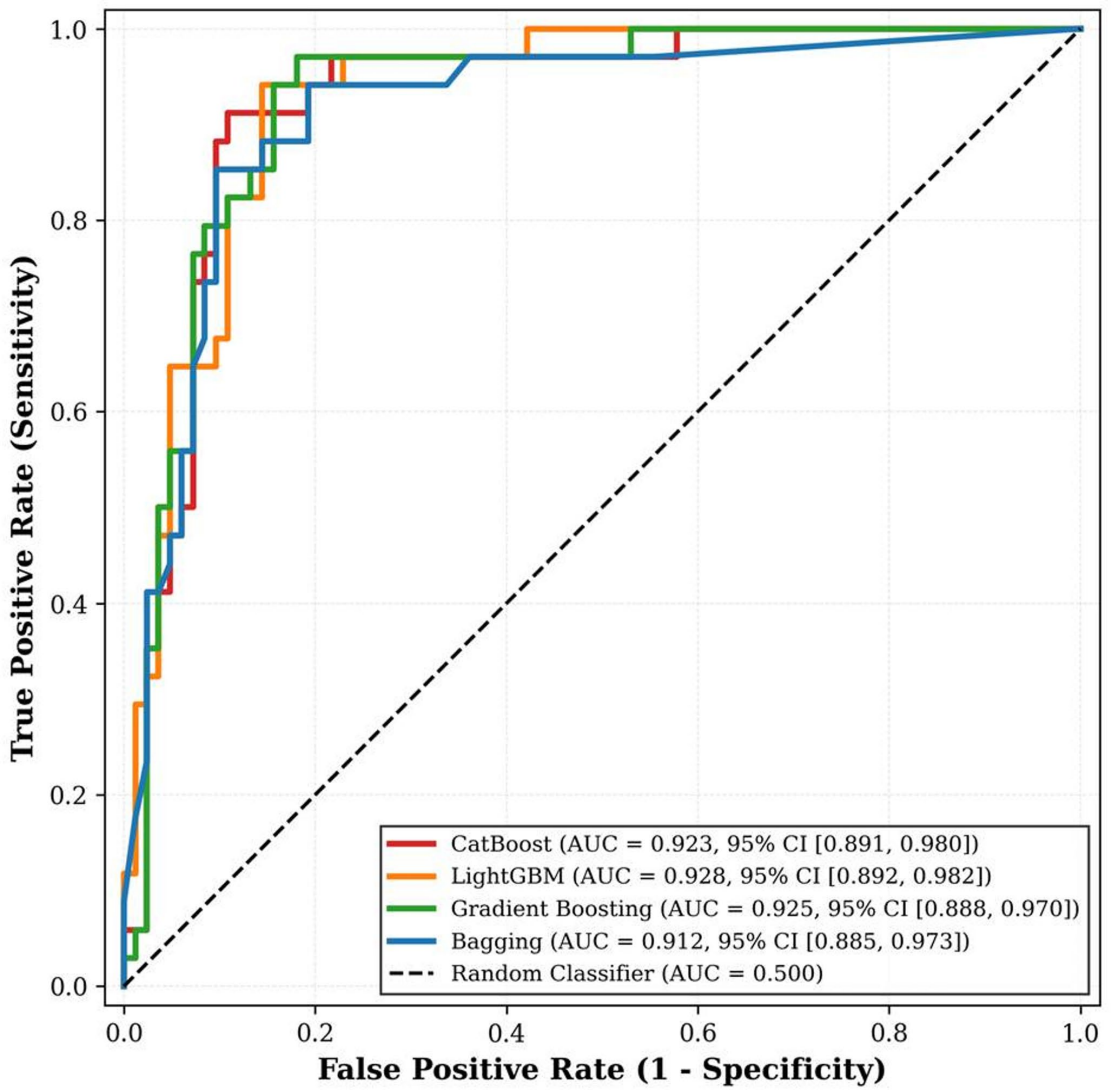
Receiver operating characteristic curves: comparison of machine learning model

The 10-fold cross-validation results are presented in Table 2. CatBoost demonstrated superior balanced performance with the highest accuracy, MCC, and F1-score. LightGBM achieved a marginally higher AUC value; however, this difference was not statistically significant as the confidence intervals overlapped considerably (*p* > 0.05). All four models demonstrated discriminative ability (AUC > 0.90). ROC curves illustrate the performance of all models across classification thresholds. CatBoost and LightGBM exhibited nearly identical ROC curves, both substantially exceeding the random classifier (Fig. 1).

**Table 2:** Performance comparison of machine learning models for testicular torsion classification

Model	Accuracy [95% CI]	AUC [95% CI]	Sensitivity [95% CI]	Specificity [95% CI]	F1-Score	MCC
CatBoost	0.895 [0.826, 0.964]	0.923 [0.891, 0.980]	0.883 [0.788, 0.978]	0.900 [0.798, 1.002]	0.847	0.796
LightGBM	0.863 [0.810, 0.916]	0.928 [0.892, 0.982]	0.833 [0.717, 0.950]	0.878 [0.805, 0.951]	0.782	0.706
Gradient Boosting	0.870 [0.805, 0.936]	0.925 [0.888, 0.970]	0.767 [0.658, 0.875]	0.912 [0.816, 1.009]	0.784	0.721
Bagging	0.869 [0.793, 0.944]	0.912 [0.885, 0.973]	0.800 [0.650, 0.950]	0.900 [0.805, 0.995]	0.783	0.717

AUC = Area Under the Receiver Operating Characteristic Curve; MCC = Matthews Correlation Coefficient; CI = Confidence Interval. Models were evaluated using 10-fold stratified cross-validation. SVMSMOTE was applied only to training sets to address class imbalance; test sets were not altered. The best-performing model (CatBoost) is highlighted in gray

The confusion matrix analysis in Supplementary Table S4 demonstrates CatBoost’s performance across both outcome classes. The PPV value indicates that the model was correct 85.6% of the time when predicting orchiectomy, while the high NPV value indicates 95.5% accuracy when predicting detorsion. These predictive values support the clinical utility of the model for surgical decision support.

### Feature importance and model interpretability with explainable artificial intelligence

Considering the highest MCC value (a critical indicator of balanced performance in imbalanced datasets), CatBoost was selected as the optimal model for subsequent interpretability analysis.

The explainable artificial intelligence analysis conducted using SHAP, LIME, model-based, and permutation-based methods is presented in Table 3.

**Table 3:** Feature Importance Comparison (SHAP, LIME, Model-Based)

Feature	SHAP (%)	LIME (%)	Model-Based (%)	Permutation – Based (%)	SHAP Rank	LIME Rank	Model Based Rank	Permutation Rank
Symptom Duration	52.72%	39.60%	54.29%	62.57%	1	1	1	1
MLR	14.24%	15.48%	6.80%	13.41%	2	3	4	2
PDW	12.09%	21.05%	12.95%	11.45%	3	2	2	3
Age	8.97%	11.14%	9.46%	1.68%	4	4	3	7
PCT	4.83%	3.71%	4.88%	2.23%	5	7	7	6
SII	4.48%	5.06%	6.57%	2.79%	6	5	5	5
MPV	2.67%	3.96%	5.05%	5.87%	7	6	6	4

SHAP, SHapley Additive exPlanations; LIME, Local Interpretable Model-agnostic Explanations; MLR, monocyte-to-lymphocyte ratio; PDW, platelet distribution width; PCT, plateletcrit; SII, systemic immune-inflammation index; MPV, mean platelet volume. Importance values represent the relative contribution (%) of each feature to model predictions. Rankings indicate the order of importance for each method (1 = most important). Symptom Duration demonstrated the highest importance across all four methods with consistent rank of 1, indicating high cross-method agreement (σ < 1.0). PDW and MLR showed moderate agreement (1.0 ≤ σ < 2.0) with rankings varying between 2–4 across methods.

**Fig. 2:**
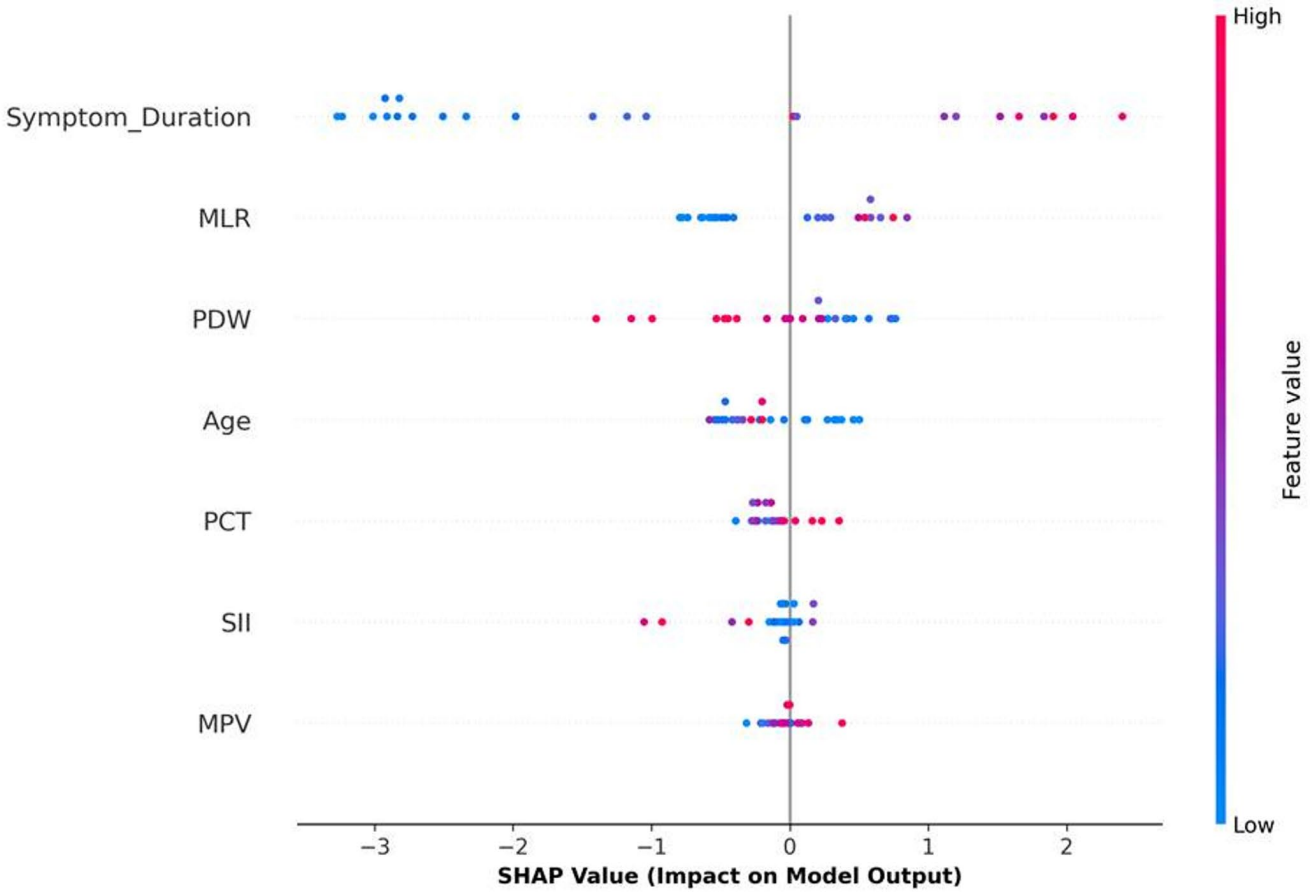
SHAP summary plot: feature impact ontesticular torsion orchiectomy risk prediction

The SHAP summary plot demonstrates in detail how feature values influence predictions. High symptom duration values were consistently associated with positive SHAP values (orchiectomy prediction), while low values were associated with negative SHAP values (detorsion prediction) (Fig. 2). This pattern is consistent with the clinical knowledge that prolonged ischemia duration reduces the probability of testicular viability.

No features demonstrated low consistency (σ ≥ 2.0), suggesting that our feature importance findings are robust and not artifacts of any particular methodology.

PDP analysis revealed feature-risk relationships. Symptom duration showed the strongest effect (risk range: 0.013→0.731, Δ = 0.718), exhibiting a distinct inflection point at approximately 7 h (0–7 h: rapid increase, 7 + h: plateau). PDW demonstrated a strong inverse relationship (Δ = 0.209) with a sharp decline at 17.9 fL. MLR exhibited a monotonically increasing pattern (Δ = 0.155) with risk escalation at 0.29. These findings corroborated the ROC cut-off values.

Bivariate PDP visualized the strongest interactions: Symptom Duration × MLR (risk range: 0.921) and Symptom Duration × PDW (0.895). The combination of prolonged symptoms and low PDW demonstrated > 85% risk, while short symptoms combined with high PDW was associated with < 10% risk, demonstrating a strong synergistic effect.

ICE analysis identified high heterogeneity for all features (score > 0.36). PDW and MLR exhibited the highest heterogeneity (0.398), reflecting patient-specific effects through non-parallel curves. These findings indicate that feature effects vary individually and support the necessity for personalized risk assessment. The analysis graphs are shown in Supplementary Figure S2.

### Nomogram development and validation

**Fig. 5:**
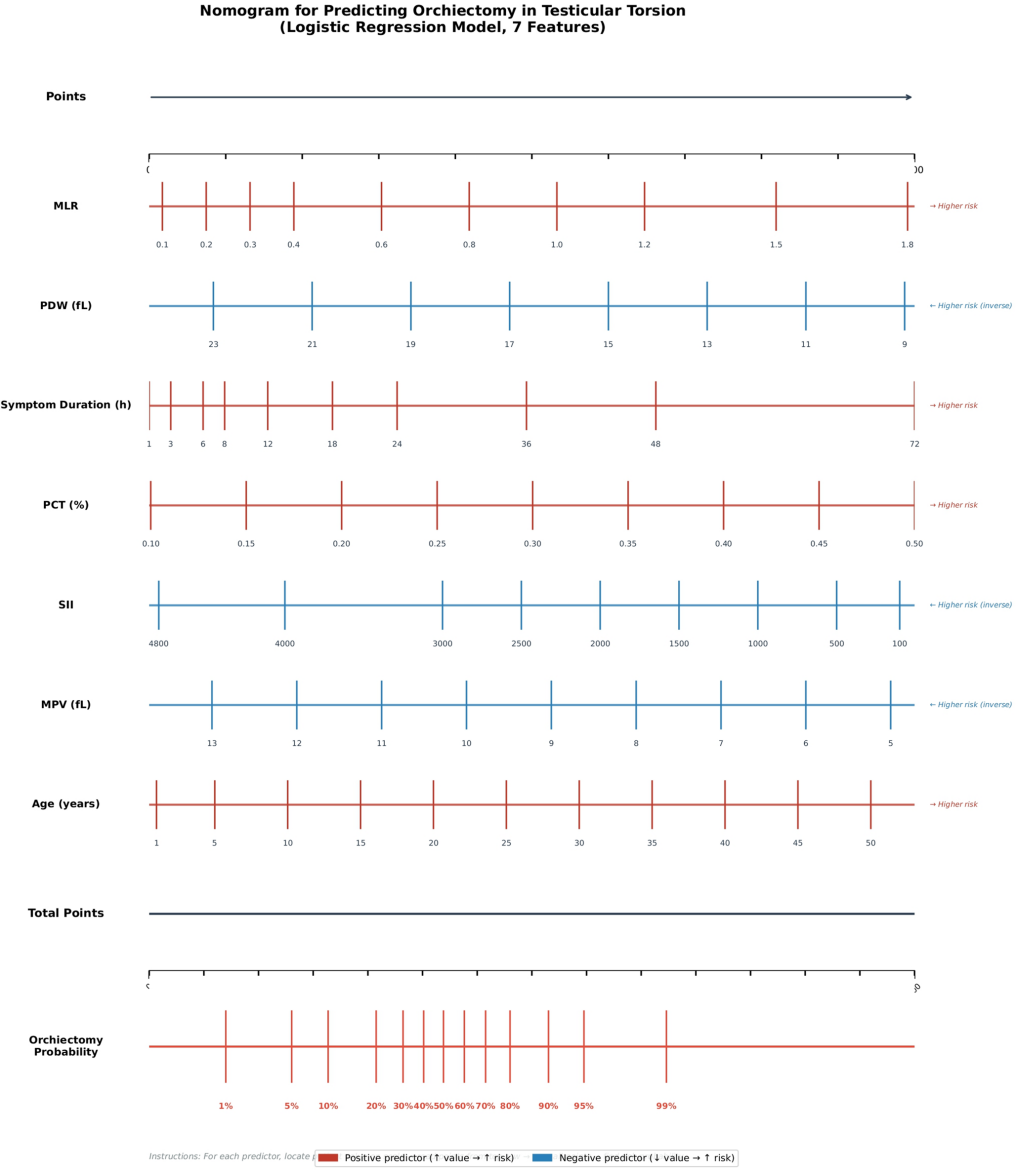
Static nomogram for predicting orchiectomy in testicular torsion. The nomogram was developed using logistic regression with the seven PSO-GWO-selected features. For each predictor, the patient’s value is located on the corresponding scale to determine the points (0–100) from the top reference line. Red scales indicate positive predictors (higher value → higher risk); blue scales indicate inverse predictors (lower value → higher risk). All feature points are summed to obtain the total score (0–700), which is then mapped to the predicted orchiectomy probability on the bottom scale

A comprehensive nomogram incorporating the seven selected features (Age, Symptom Duration, PDW, PCT, MPV, MLR, SII) was developed for bedside use in clinical practice. Using a logistic regression model, a scale ranging from 0 to 100 points was created for each feature, with a total score calculated in the range of 0–700. Regression coefficient analysis revealed that MLR was the strongest positive predictor (β=+0.765) and PDW was the strongest negative predictor (β=−0.595). Symptom Duration (β=+0.346) and PCT (β=+0.328) provided moderate positive contributions, while Age (β=+0.019) showed minimal effect (Table 4). Nomogram discrimination performance was good with an AUC of 0.818 (95% CI 0.742–0.894). Model accuracy was calculated as 82.1%, sensitivity as 79.4%, and specificity as 83.1%. Calibration analysis demonstrated excellent fit (Hosmer-Lemeshow test: χ²=6.82, *p* = 0.556; Brier score: 0.165; calibration slope: 0.94, CI: 0.81–1.07). The calibration curve showed that observed and predicted probabilities followed closely to the 45° ideal line. The traditional static nomogram for bedside use without internet access is presented in Fig. 5.

**Table 4:** Nomogram logistic regression coefficients and point ranges

Feature	β Coefficient (Std. Error)	Feature Range	Point Range
MLR	+ 0.765 (0.183)	0.07–1.82	0–100
PDW*	−0.595 (0.162)	8.8–24.3 fL	100–0
Symptom Duration	+ 0.346 (0.141)	1–72 h	0–100
PCT	+ 0.328 (0.138)	0.10–0.50%	0–100
SII*	−0.232 (0.127)	6–4861	100–0
MPV*	−0.212 (0.119)	4.7–13.7 fL	100–0
Age	+ 0.019 (0.098)	0.5–53 years	0–100
	Intercept (β₀): −1.034 (0.267)		Total Points Range: 0–700

* Inverse relationship: Lower value = higher points (risk factor) β coefficient magnitudes: |β| > 0.5 (strong), 0.3–0.5 (moderate), < 0.3 (weak) All coefficients are statistically significant (*p* < 0.05)

### Interactive web application for testicular torsion surgical risk prediction

**Fig. 3:**
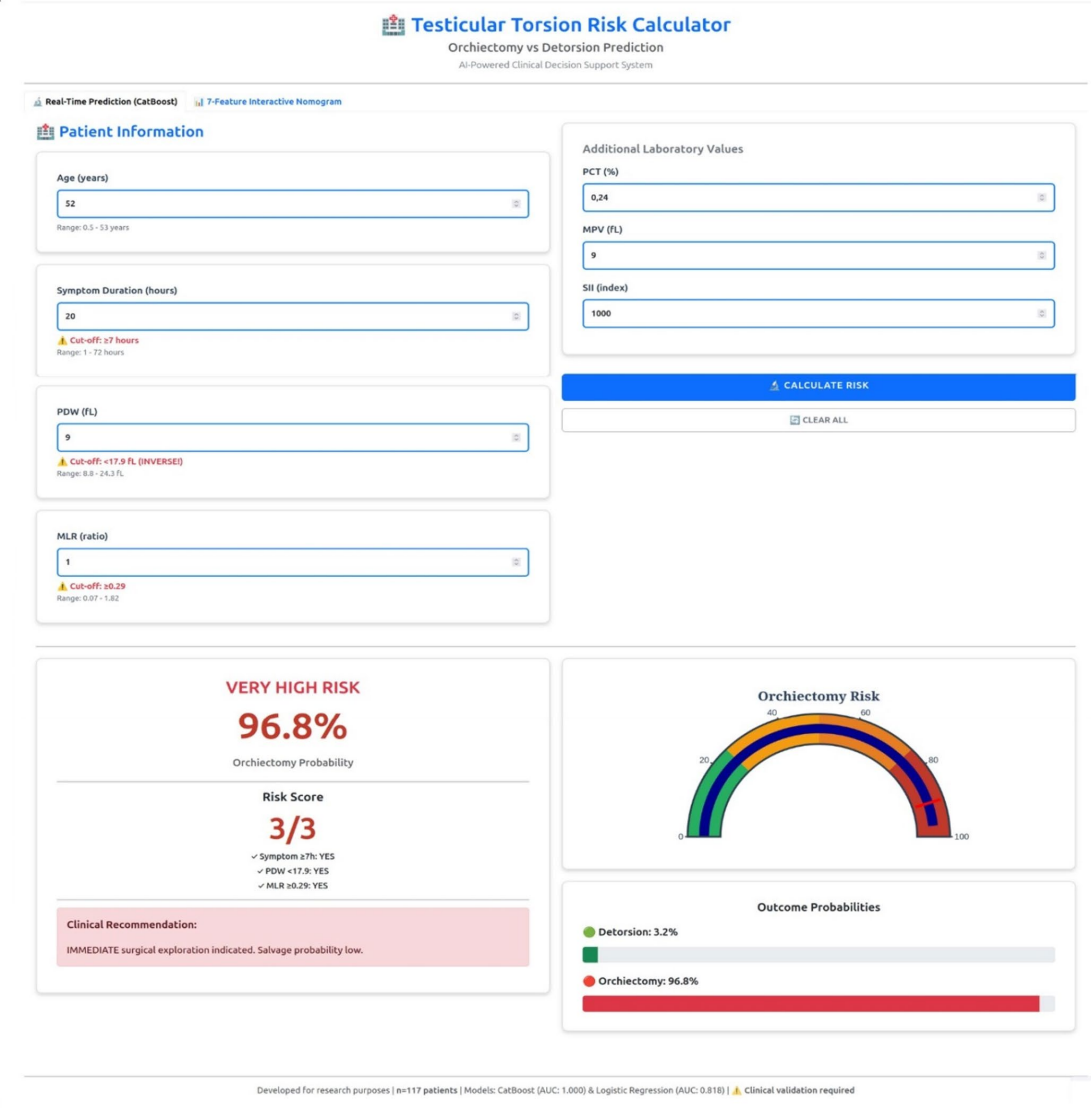
A web interface that receives patient parameters and generates the orchiectomy risk percentage and risk level

**Fig. 4:**
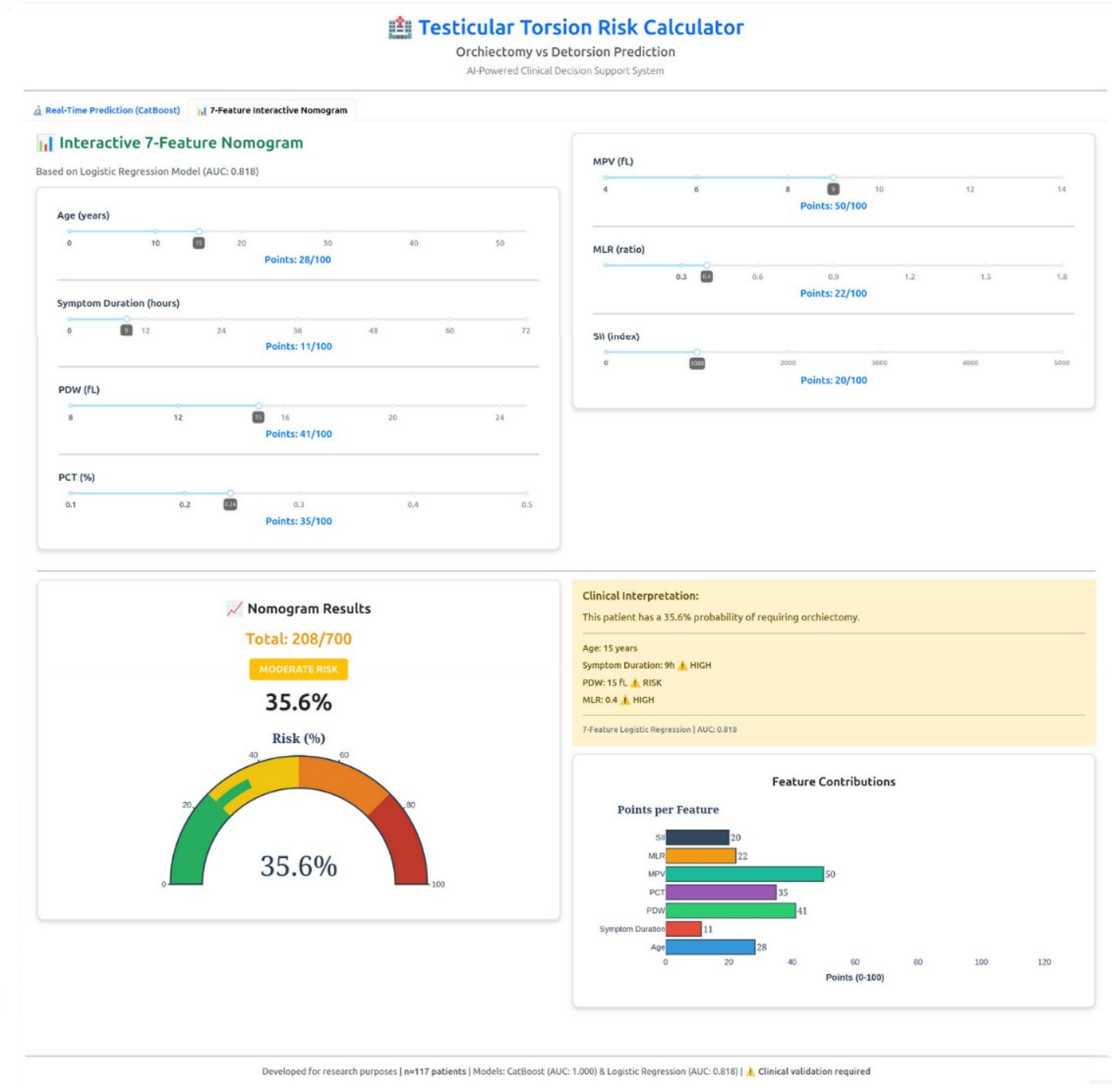
Interactive nomogram module showing feature points, total score, and predicted orchiectomy risk

A two-module web application was created to ensure clinical applicability of the developed models (medicalinformaticsttrc.adiyaman.edu.tr). The first module (CatBoost Real-Time Prediction) receives patient parameters and generates risk percentage, risk level (Low/Moderate/High/Very High), a risk score ranging from 0 to 3, and clinical recommendations in < 50 ms. The second module (Interactive 7-Feature Nomogram) provides instant point calculation for each feature (0–100), total score (0–700), and risk percentage through slider-based input. The application operates securely with HTTPS encryption without storing patient data and is freely accessible 24/7 (Figs. 3 and 4).

## Discussion

Artificial intelligence (AI) refers to the computational ability of machines to mimic and perform human cognitive tasks. It is causing a paradigm shift in healthcare delivery and decision-making for clinical practitioners. Advances in medical technologies used in healthcare, such as electronic medical records, are providing enormous amounts of data. This large volume of data enables computer-based predictions and decisions to be made to assist in better patient care [10]. AI is widely used in the diagnosis, treatment, and outcome prediction of various urological diseases. In all benign conditions, it has been applied to predict surgical procedure outcomes, stone composition in urolithiasis, and disease severity in pediatric urology and BPH; in malignant conditions, it has been used to predict treatment response, survival, prognosis, and recurrence based on genomic and biomarker studies. These outcomes have also been found to be statistically better than conventional approaches [11].

The orchiectomy rate in cases with TT is 32% [12]. Similarly, in our study, it was 29.1%. Accurate preoperative planning is important because complications such as infection and anti-sperm antibody-mediated infertility can be prevented by orchiectomy in cases of non-viable necrotic testis [13]. Determining testicular viability after TT is a major challenge in clinical practice. There are studies using conventional statistical methods analyzing the effects of various factors on testicular viability. Some of these studies have shown that testicular viability is significantly associated with symptom duration and degree of torsion [5, 14]. It has been reported that testicular viability can be preserved in 90–100% of TT cases when surgical intervention is performed within the first 6 h, and this rate decreases to 50% when performed after 12 h [15]. On the other hand, it is known that not all TT cases explored after 12 h require orchiectomy, and testicular volume loss may occur in the first 6-hour period in 44% of surgically treated cases [13]. Studies on hematological parameters for determining testicular viability after TT have been reported. Jang et al. stated that NLR, in addition to symptom duration and degree of torsion, was significantly associated with testicular viability [6]. Furthermore, studies by Benlioğlu et al. [16] and Ekşi et al. [9] reported that monocyte count was significantly higher in the orchiectomy group. The functions of monocytes in the immune system include cytokine expression, antigen presentation, and phagocytosis. It has been shown that abnormally activated monocytes may play a role in the pathogenesis of inflammation [17]. Considering that intact, undamaged testicular tissues are naturally protected from the immunological system, it has been hypothesized that histological and physiological changes in torsioned and damaged testicular tissues may cause a monocyte reaction [9].

Nomograms, which combine multiple clinical variables in a simplified table, are widely used in medical research. Chen et al., using multivariate logistic regression analysis among conventional statistical methods, identified symptom duration, intratesticular blood flow, degree of spermatic cord torsion, and monocyte count as independent risk factors for testicular salvage after TT. They constructed a nomogram based on these four risk factors and used the validation cohort data for ROC analysis. They found an AUC of 0.965 (95% CI 0.867–0.997), sensitivity of 90.5%, and specificity of 88.9%. It was reported that this prediction model could provide parents and clinicians of children with TT with a precise probability based on the child’s testicular salvage status [4].

Mao et al. constructed a nomogram to predict the probability of orchiectomy in children with TT based on the results of multivariate logistic regression analysis. They reported that the nomogram, by assigning a score to each value of the variables, allowed clinicians to quickly assess risk by vertically matching the total score of all variables with the estimated probability of orchiectomy, and that the higher the total score, the higher the risk of orchiectomy. They reported that the constructed nomogram exhibited excellent discriminative ability with AUC values of 0.93 and 0.86 in the training and validation cohorts, respectively, indicating satisfactory predictive performance [18].

Artificial intelligence studies to predict the probability of orchiectomy using preoperative parameters in patients presenting with TT are quite limited. In their study comparing models developed using classical statistical methods and ML models to predict the probability of orchiectomy using preoperative parameters in patients presenting with TT, Ekşi et al. found the sensitivity and specificity of the model created with conventional statistical methods to be 88% and 87%, and the sensitivity and specificity of the model created with ML to be 92% and 89%, respectively, and reported that the ML model performed better than the traditional statistical regression model in predicting orchiectomy [9].

Artificial intelligence and machine learning techniques hold great potential for resolving complex relationships in biomedical data. However, the greatest barrier to the clinical translation of these models is the lack of transparency in their decision-making processes, making them “black boxes.” SHAP and similar explainable AI approaches proposed by Tonekaboni et al. demonstrate how much weight the model assigns to which variables and enhance clinical interpretability [19].

### Clinical utility and intended use

It is important to emphasize that the developed prediction model and web application are not intended to influence the decision to perform emergent scrotal exploration, which remains mandatory upon clinical suspicion of testicular torsion. Rather, the tools serve three complementary clinical purposes: (1) preoperative counseling—enabling clinicians to provide families with quantitative risk estimates to set realistic expectations regarding potential testicular loss; (2) surgical preparedness—facilitating logistical planning including prosthetic implant availability, anticipated operative duration, and postoperative fertility counseling for high-risk patients; and (3) medicolegal documentation—providing objective, quantifiable risk stratification documented prior to surgery.

### Clinical Interpretation of Selected Features

Symptom duration emerged as the dominant predictor across all four XAI methods (SHAP: 52.72%). The PDP analysis revealed a distinct inflection point at approximately 7 h, providing a data-driven validation of the classical “6-hour golden window.” This inflection point corroborates clinical observations that irreversible ischemic injury accelerates beyond 6–8 h and can serve as a practical threshold for risk communication with families.

The inverse association between PDW and orchiectomy risk (lower PDW → higher risk; SHAP: 12.09%) is a novel finding with biological plausibility. PDW reflects platelet size heterogeneity, which decreases during acute inflammatory states due to increased consumption of large, reactive platelets in the microcirculation. In testicular ischemia, microvascular platelet activation and aggregation may selectively deplete larger platelets, thereby reducing PDW. Crucially, PDW is a practical, cost-free biomarker available from routine CBC within 5 min, requiring no additional laboratory tests.

Elevated MLR (SHAP: 14.24%) reflects monocyte activation in response to ischemic tissue damage. Monocytes are recruited to ischemic testicular tissue as part of the innate immune response, consistent with prior reports demonstrating elevated monocyte counts in the orchiectomy group [9, 16]. The threshold of 0.29 identified by PDP analysis may serve as a simple bedside criterion for risk stratification.

**Fig. 6:**
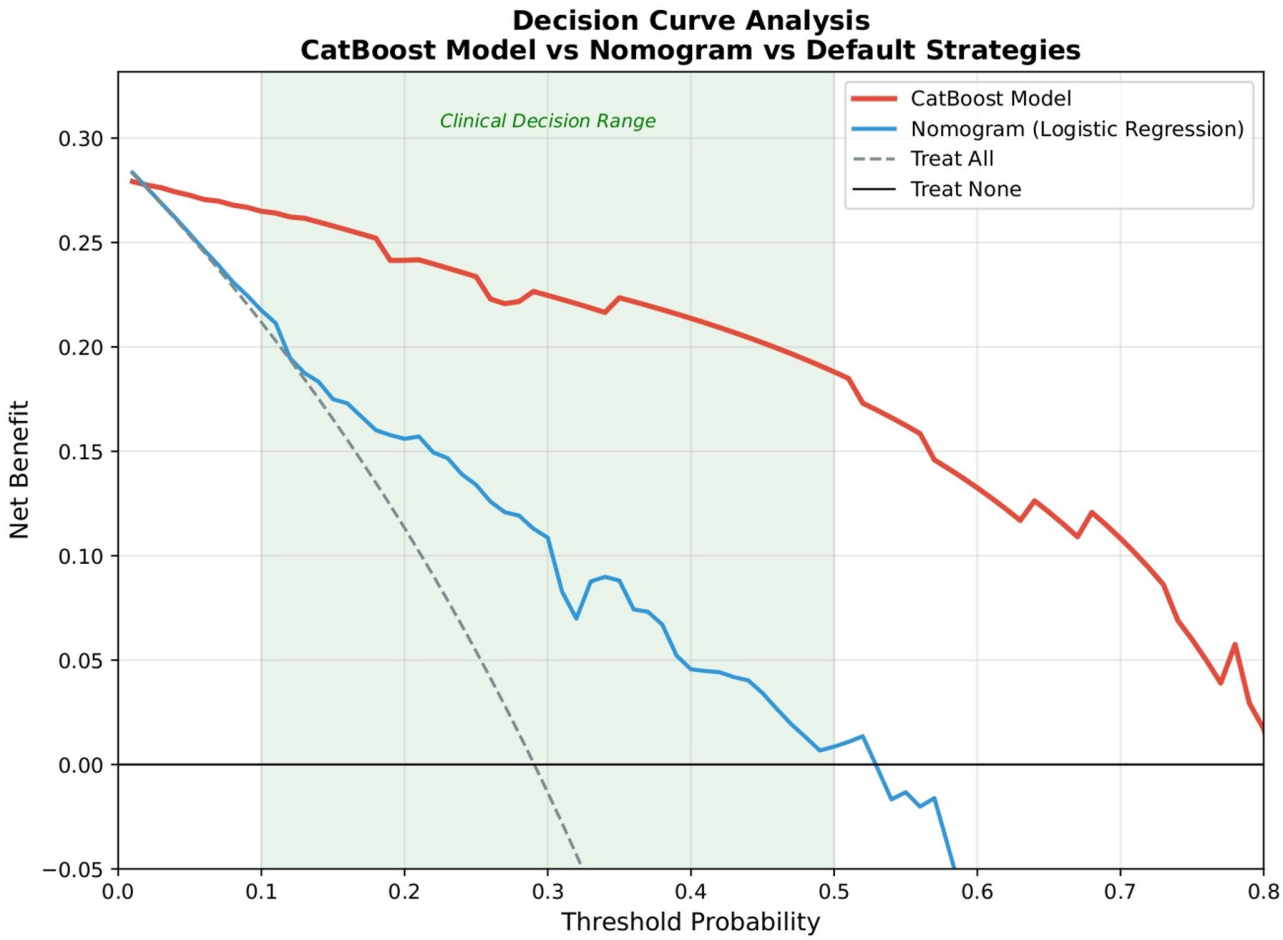
Decision curve analysis comparing the CatBoost model, nomogram (logistic regression), “Treat All,” and “Treat None” strategies. The x-axis represents the threshold probability at which a clinician would consider intervention, and the y-axis represents the net benefit. The CatBoost model demonstrated positive net benefit across the clinically relevant threshold range (0.10–0.80), consistently outperforming both the nomogram and default strategies. The shaded region indicates the clinical decision range

For bedside applicability, the simplified 3-feature risk score (Symptom Duration > 7 h = 1 point, PDW < 17.9 fL = 1 point, MLR > 0.29 = 1 point) offers a practical tool that can be calculated mentally in seconds: score 0–1 indicates low risk (~ 0–5%), score 2 indicates moderate risk (~ 44%), and score 3 indicates high risk (100%). Decision curve analysis demonstrated that the CatBoost model achieved positive net benefit across the entire clinically relevant threshold range (0.10–0.80), outperforming both the nomogram and default treat-all/treat-none strategies (Fig. 6). Threshold-specific performance metrics are presented in Table S1; at a screening threshold of 0.10, the model achieved 97.1% sensitivity with 98.5% NPV, while the Youden optimal threshold (0.511) balanced sensitivity (91.2%) and specificity (89.2%).

### Novel aspects of our study

This study is the first comprehensive artificial intelligence study with several novel aspects in predicting orchiectomy after testicular torsion. First, optimal feature selection was performed using the hybrid PSO-GWO algorithm. Second, systematic application of explainable artificial intelligence (XAI) techniques (SHAP, LIME, PDP, ICE) was performed. Third, both a high-performance ML model (CatBoost, AUC: 0.923) and a clinically usable nomogram (AUC: 0.818) were developed. Fourth, an interactive web-based decision support system was created. Fifth, PDW was characterized as a novel prognostic biomarker in testicular torsion.

### Methodological contributions

Nine methods were compared for feature selection, with hybrid PSO-GWO demonstrating the highest performance (MCC: 0.705 ± 0.092). Eight resampling strategies were evaluated for class imbalance, with SVMSMOTE found to be superior (MCC: 0.713). Five different XAI techniques were applied. PDP and ICE analyses revealed nonlinear relationships and feature interactions: an inflection point at 7 h for symptom duration, an inverse monotonic relationship for PDW, and a Symptom × PDW synergistic effect were identified. The high heterogeneity in ICE analysis (> 0.36) emphasized the necessity for patient-specific evaluation.

### Clinical Contributions

The study provided three important tools for clinical practice: (1) A simple 3-feature risk score (0–3 points), applicable at the bedside; (2) A seven-feature nomogram with excellent calibration (Hosmer-Lemeshow *p* = 0.556) for reliable risk estimation; (3) A web application for real-time risk calculation (< 500 ms) and clinical decision support. XAI analyses confirmed that the model produces biologically plausible predictions, enhancing clinical acceptability. PDW was identified as a novel biomarker. While NLR and PLR, which are widely used in the literature, did not show significant differences in our study (p_FDR>0.55), PDW demonstrated strong discriminatory power (Δ = 0.209, SHAP: 12.09%). This finding is consistent with a recent meta-analysis reporting inconsistent NLR performance across TT cohorts (14), and reflects the PSO-GWO algorithm’s ability to identify that MLR and SII subsumed the predictive information carried by NLR and PLR. The advantages of PDW include: measurement at no additional cost in routine CBC, results in < 5 min, and being objective and reproducible.

In summary, our study is the first to integrate hybrid metaheuristic feature selection (PSO-GWO), ensemble machine learning (CatBoost), comprehensive XAI techniques (SHAP, LIME, PDP, ICE), a logistic regression-based nomogram, and an interactive web-based decision support system for predicting orchiectomy in patients with TT.

### Study limitations

The main limitations are the retrospective design, small sample size (*n* = 117), and single-center cohort. With 34 orchiectomy events and seven predictor features, the events-per-variable ratio is approximately 4.9, which is below the commonly recommended minimum of 10 EPV, increasing the risk of model overfitting. Therefore, this model should be considered exploratory and requires external validation in independent, preferably multicenter cohorts of > 500 patients before clinical implementation. External validation was not performed, and model generalizability is uncertain. Class imbalance (2.44:1) was addressed with SVMSMOTE but may not fully reflect the diversity of real patients. Ultrasound findings, surgical details, and additional laboratory parameters were not included in the model. The prognostic value of PDW was identified for the first time, and its independent validation and pathophysiological mechanism should be elucidated. The real-world use of the web application in clinical settings should be evaluated.

Another significant limitation of our study is the lack of postoperative follow-up data (testicular atrophy, volume loss, or functional outcome) for our patients. The “detorsion” result only indicates that the testis was not removed during surgery; it does not confirm long-term salvage. Therefore, it would be appropriate to conduct similar studies with patients who underwent postoperative follow-up to discuss testicular salvage surgery. Prospective, multicenter studies are essential to validate these findings.

## Conclusion

The model developed with hybrid PSO-GWO and CatBoost achieved high accuracy in predicting orchiectomy in testicular torsion (AUC: 0.923, MCC: 0.796). XAI analyses identified symptom duration as the strongest predictor (SHAP: 52.72%) and PDW as a novel biomarker. The nomogram, risk score, and web application provided practical tools for preoperative counseling, surgical preparedness, and clinical decision support. It should be noted that the “detorsion” outcome reflects intraoperative non-removal and does not confirm long-term testicular viability. PDW is a practical and powerful biomarker candidate that can be measured at no additional cost in routine CBC. However, this exploratory model requires external validation in multicenter prospective cohorts, and mechanistic investigations of PDW are warranted before clinical implementation.

Future research directions include: (1) External validation in larger cohorts (> 500 patients), (2) Extended feature sets with ultrasound and surgical findings, (3) Elucidation of the pathophysiological mechanism of PDW, (4) Age-specific models, and (5) Clinical impact analysis of the web application. This study highlights the potential of artificial intelligence in medical decision support systems and the importance of digital transformation in clinical medicine.

## Supplementary Information

Below is the link to the electronic supplementary material.

Supplementary Material 1

## Data Availability

The data sets generated during and/or analyzed during the current study are available from the corresponding author upon reasonable request.

## Abbreviations

ADASYN: Adaptive synthetic sampling
AUC: Area under the curve
BGOA: Binary grasshopper optimization algorithm
CBC: Complete blood count
CI: Confidence interval
FDR: False discovery rate
FN: False negative
FP: False positive
HL: Hosmer–Lemeshow
ICE: Individual conditional expectation
LR: Logistic regression
LIME: local interpretable model–agnostic explanations
MCC: Matthews correlation coefficient
ML: Machine learning
MLR: Monocyte–to–lymphocyte ratio
MPV: Mean platelet volume
NLR: Neutrophil–to–lymphocyte ratio
NPV: Negative predictive value
NRS: Neighborhood rough set
PCT: Plateletcrit
PDP: Partial dependence plot
PDW: Platelet distribution width
PIV: Pan–immune–inflammation value
PPV: Positive predictive value
PSO–GWO: Particle swarm optimization–grey wolf optimization
ROC: Receiver operating characteristic
RFE: Recursive feature elimination
RDW: Red cell distribution width
SHAP: SHapley Additive exPlanations
SII: Systemic immune–inflammation index
SMOTE: Synthetic minority over–sampling technique
SVMSMOTE: Support vector machine–based SMOTE
TN: True negative
TP: True positive
TT: Testicular torsion
TWIST: Testicular workup for ischemia and suspected torsion
US: Ultrasonography
XAI: Explainable artificial intelligence
