# Supplementary material for: Predicting orchiectomy in testicular torsion using hybrid machine learning and explainable AI: a web-based clinical decision support system
Source: World J Urol. 2026 Jun 8;44(1):414. doi: 10.1007/s00345-026-06448-4 (PMC13246560; doi:10.1007/s00345-026-06448-4)
Supplement: Supplementary file 1 — Supplementary Material 1 [file 345_2026_6448_MOESM1_ESM.docx]

**Table S1.** CatBoost Model Performance Metrics Across Classification Thresholds (Pooled 10-Fold Cross-Validation)

| **Threshold** | **Sensitivity** | **Specificity** | **PPV** | **NPV** | **Accuracy** | **F1-Score** | **TP** | **FP** | **FN** | **TN** |
| --- | --- | --- | --- | --- | --- | --- | --- | --- | --- | --- |
| 0.10 | 0.971 | 0.783 | 0.647 | 0.985 | 0.838 | 0.776 | 33 | 18 | 1 | 65 |
| 0.15 | 0.971 | 0.807 | 0.673 | 0.985 | 0.855 | 0.795 | 33 | 16 | 1 | 67 |
| 0.20 | 0.941 | 0.819 | 0.681 | 0.971 | 0.855 | 0.790 | 32 | 15 | 2 | 68 |
| 0.25 | 0.941 | 0.831 | 0.696 | 0.972 | 0.863 | 0.800 | 32 | 14 | 2 | 69 |
| 0.30 | 0.912 | 0.867 | 0.738 | 0.960 | 0.880 | 0.816 | 31 | 11 | 3 | 72 |
| 0.35 | 0.912 | 0.892 | 0.775 | 0.961 | 0.897 | 0.838 | 31 | 9 | 3 | 74 |
| 0.40 | 0.912 | 0.892 | 0.775 | 0.961 | 0.897 | 0.838 | 31 | 9 | 3 | 74 |
| 0.45 | 0.912 | 0.892 | 0.775 | 0.961 | 0.897 | 0.838 | 31 | 9 | 3 | 74 |
| **0.50*** | **0.912** | **0.892** | **0.775** | **0.961** | **0.897** | **0.838** | **31** | **9** | **3** | **74** |
| 0.55 | 0.882 | 0.892 | 0.769 | 0.949 | 0.889 | 0.822 | 30 | 9 | 4 | 74 |
| 0.60 | 0.853 | 0.892 | 0.763 | 0.937 | 0.880 | 0.806 | 29 | 9 | 5 | 74 |
| 0.65 | 0.853 | 0.904 | 0.784 | 0.938 | 0.889 | 0.817 | 29 | 8 | 5 | 75 |
| 0.70 | 0.853 | 0.916 | 0.806 | 0.938 | 0.897 | 0.829 | 29 | 7 | 5 | 76 |
| 0.75 | 0.824 | 0.916 | 0.800 | 0.927 | 0.889 | 0.812 | 28 | 7 | 6 | 76 |
| 0.80 | 0.765 | 0.928 | 0.812 | 0.906 | 0.880 | 0.788 | 26 | 6 | 8 | 77 |

**Note.** PPV = Positive Predictive Value; NPV = Negative Predictive Value; TP = True Positive; FP = False Positive; FN = False Negative; TN = True Negative. All metrics were calculated from pooled predictions across 10-fold stratified cross-validation (n = 117). SVMSMOTE was applied only to training folds; test folds remained unaltered.

* Youden optimal threshold (maximizing sensitivity + specificity − 1). The threshold of 0.511 was rounded to 0.50 for the table.

Highlighted row (★) indicates the Youden optimal threshold.

***Clinical interpretation:*** *At a low-risk screening threshold (0.10), sensitivity of 97.1% and NPV of 98.5% ensure that virtually no orchiectomy cases are missed. At the Youden optimal threshold (0.50), balanced performance is achieved with sensitivity of 91.2% and specificity of 89.2%. At a high-specificity threshold (0.70), specificity of 91.6% and PPV of 80.6% provide high confidence in positive predictions.*

**Table S2**. Performance Comparison of Feature Selection Methods

| **Method** | **MCC (Mean ± SD)** | **N Features** | **Selected Features** |
| --- | --- | --- | --- |
| **PSO-GWO** | **0.705 ± 0.092** | **7** | **Symptom_Duration, PDW, PCT, MPV, MLR, SII, Age** |
| **CBR-BGOA** | 0.621 ± 0.147 | 6 | Symptom_Duration, RDW, Platelet_Count, PDW, Monocyte, MLR |
| **RFE** | 0.574 ± 0.166 | 7 | Symptom_Duration, PDW, PLR, Neutrophil, Monocyte, MLR, PIV |
| **BORUTA** | 0.571 ± 0.189 | 5 | Symptom_Duration, PDW, Monocyte, MLR, PIV |
| **ANOVA F-test** | 0.571 ± 0.163 | 7 | Symptom_Duration, RDW, Platelet_Count, PDW, Monocyte, MLR, PIV |
| **LASSO** | 0.552 ± 0.192 | 5 | Symptom_Duration, RDW, PDW, Monocyte, PIV |
| **Mutual Info** | 0.531 ± 0.146 | 7 | Symptom_Duration, PCT, PLR, Lymphocyte, Monocyte, MLR, PIV |
| **Chi-Square** | 0.503 ± 0.169 | 7 | Symptom_Duration, Platelet_Count, PDW, PLR, Monocyte, PIV, SII |
| **ElasticNet** | 0.476 ± 0.126 | 11 | Age, Symptom_Duration, WBC, Platelet_Count, PDW, PLR, ... |
| **NRS** | 0.447 ± 0.170 | 7 | Age, Symptom_Duration, PCT, Lymphocyte, Monocyte, MLR, PIV |

**Note.**MCC = Matthews Correlation Coefficient; SD = Standard Deviation; N Features = Number of selected features; PSO-GWO = Particle Swarm Optimization-Grey Wolf Optimizer; CBR-BGOA = Case-Based Reasoning with Binary Grasshopper Optimization Algorithm; RFE = Recursive Feature Elimination; NRS = Neighborhood Rough Set. All methods evaluated using 5-fold stratified cross-validation. Best performing method (PSO-GWO) shown in bold.

**Table S3.** Performance Comparison of Synthetic Data Generation Methods

| **Method** | **MCC** | **Accuracy** | **Precision** | **Sensitivity** | **Specificity** | **F1** | **AUC-ROC** |
| --- | --- | --- | --- | --- | --- | --- | --- |
| **SVMSMOTE** | **0.713** | **0.875** | **0.750** | **0.857** | **0.882** | **0.800** | **0.908** |
| **Random Oversampling** | 0.597 | 0.833 | 0.714 | 0.714 | 0.882 | 0.714 | 0.916 |
| **SMOTE** | 0.597 | 0.833 | 0.714 | 0.714 | 0.882 | 0.714 | 0.882 |
| **BorderlineSMOTE** | 0.597 | 0.833 | 0.714 | 0.714 | 0.882 | 0.714 | 0.899 |
| **ADASYN** | 0.597 | 0.833 | 0.714 | 0.714 | 0.882 | 0.714 | 0.891 |
| **SMOTE-ENN** | 0.597 | 0.833 | 0.714 | 0.714 | 0.882 | 0.714 | 0.866 |
| **SMOTE-Tomek** | 0.597 | 0.833 | 0.714 | 0.714 | 0.882 | 0.714 | 0.891 |
| **No Sampling(Baseline)** | 0.476 | 0.792 | 0.667 | 0.571 | 0.882 | 0.615 | 0.924 |

**Note.**MCC = Matthews Correlation Coefficient; AUC-ROC = Area Under Curve - Receiver Operating Characteristic; SMOTE = Synthetic Minority Over-sampling Technique; ADASYN = Adaptive Synthetic Sampling; ENN = Edited Nearest Neighbors. All metrics are calculated on the original (unmanipulated) test set (n = 24). The best-performing method (SVMSMOTE) is shown in bold. No sampling strategy was applied in the baseline model.

**Table S4.** Confusion Matrix–Based Performance Metrics for Testicular Torsion Classification

| **Model** | **TP (%)** | **TN (%)** | **FP (%)** | **FN (%)** | **PPV  [95% CI]** | **NPV  [95% CI]** | **Accuracy** |
| --- | --- | --- | --- | --- | --- | --- | --- |
| **CatBoost** | 30 (88.2%) | 75 (90.4%) | 8 (9.6%) | 4 (11.8%) | 0.856  [0.729, 0.984] | 0.955  [0.919, 0.991] | 0.897 (105/117) |
| **LightGBM** | 28 (82.4%) | 73 (88.0%) | 10 (12.0%) | 6 (17.6%) | 0.775  [0.669, 0.881] | 0.931  [0.884, 0.979] | 0.863 (101/117) |
| **Gradient Boosting** | 26 (76.5%) | 76 (91.6%) | 7 (8.4%) | 8 (23.5%) | 0.856  [0.729, 0.984] | 0.913  [0.870, 0.956] | 0.872 (102/117) |
| **Bagging** | 27 (79.4%) | 75 (90.4%) | 8 (9.6%) | 7 (20.6%) | 0.815  [0.677, 0.953] | 0.923  [0.867, 0.980] | 0.872 (102/117) |

**Note:** TP = True Positive (Correct orchiectomy prediction); TN = True Negative (Correct detorsion prediction); FP = False Positive (Type I error); FN = False Negative (Missed orchiectomy – Type II error); PPV = Positive Predictive Value (Precision); NPV = Negative Predictive Value.


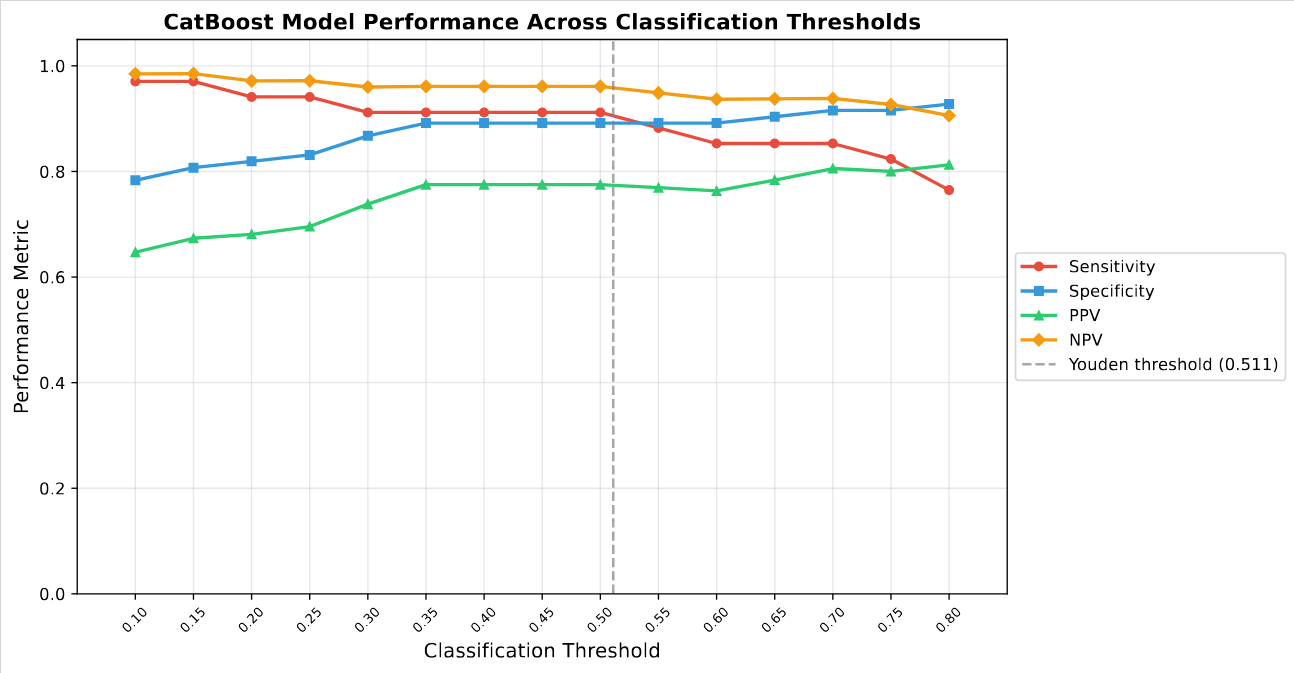


**Figure S1.** CatBoost model performance metrics across classification thresholds (pooled 10-fold cross-validation). Sensitivity (red), specificity (blue), positive predictive value (PPV, green), and negative predictive value (NPV, orange) are plotted as a function of the classification threshold (0.10–0.80). The dashed vertical line indicates the Youden optimal threshold (0.511). At lower thresholds, the model favors high sensitivity and NPV (minimizing missed orchiectomy cases), while at higher thresholds, specificity and PPV increase (maximizing confidence in positive predictions). SVMSMOTE was applied only to training folds; test folds remained unaltered.


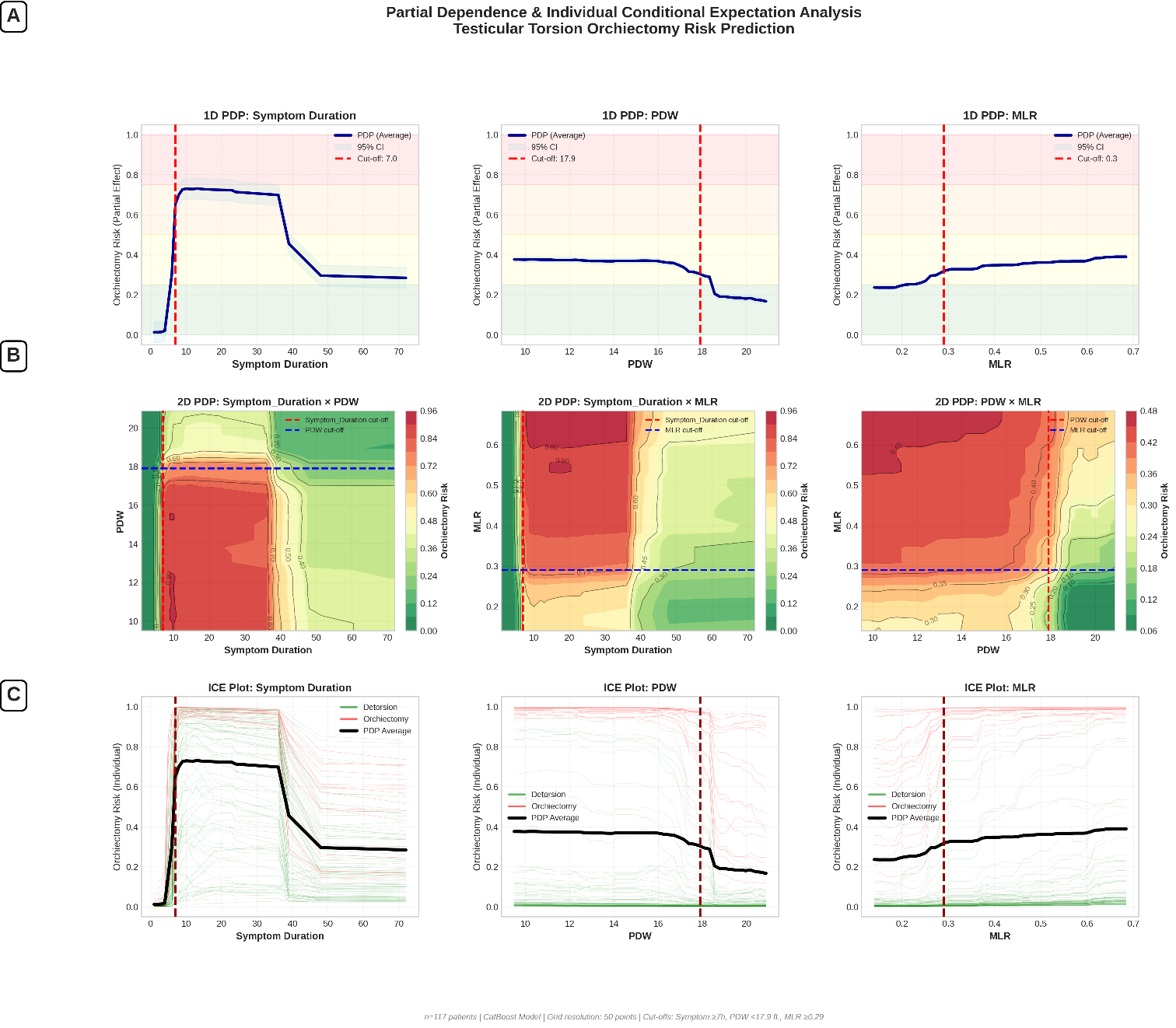


**Figure S2.** Partial Dependence – Individual Conditional Expectation Analysis Testicular Torsion Orchiectomy Risk Prediction
